# Cell Senescence: A Nonnegligible Cell State under Survival Stress in Pathology of Intervertebral Disc Degeneration

**DOI:** 10.1155/2020/9503562

**Published:** 2020-08-31

**Authors:** Yuang Zhang, Biao Yang, Jingkai Wang, Feng Cheng, Kesi Shi, Liwei Ying, Chenggui Wang, Kaishun Xia, Xianpeng Huang, Zhe Gong, Chao Yu, Fangcai Li, Chengzhen Liang, Qixin Chen

**Affiliations:** ^1^Department of Orthopedics Surgery, The Second Affiliated Hospital, Zhejiang University School of Medicine, 310009 Hangzhou, Zhejiang, China; ^2^Zhejiang Key Laboratory of Bone and Joint Precision and Department of Orthopedics Research Institute of Zhejiang University, Hangzhou, Zhejiang 310009, China

## Abstract

The intervertebral disc degeneration (IDD) with increasing aging mainly manifests as low back pain (LBP) accompanied with a loss of physical ability. These pathological processes can be preliminarily interpreted as a series of changes at cellular level. In addition to cell death, disc cells enter into the stagnation with dysfunction and deteriorate tissue microenvironment in degenerative discs, which is recognized as cell senescence. During aging, many intrinsic and extrinsic factors have been proved to have strong connections with these cellular senescence phenomena. Growing evidences of these connections require us to gather up critical cues from potential risk factors to pathogenesis and relative interventions for retarding cell senescence and attenuating degenerative changes. In this paper, we try to clarify another important cell state apart from cell death in IDD and discuss senescence-associated changes in cells and extracellular microenvironment. Then, we emphasize the role of oxidative stress and epigenomic perturbations in linking risk factors to cell senescence in the onset of IDD. Further, we summarize the current interventions targeting senescent cells that may exert the benefits of antidegeneration in IDD.

## 1. Introduction

A systematic analysis for the Global Burden of Disease Study 2017 showed that low back pain was the top cause of years lived with disability (YLD) counts from 1990 to 2017 [1]. Concerning the relation with potential loss of functional status in the work force, disc degenerative diseases, as the major cause of low back pain, have posed huge burden on the global health care systems and economies [2, 3]. Ascribed to the lifespan extension and the growth of aging population worldwide, the incidence of IDD will progressively and steadily increase and exacerbate the problem above. Multiple studies support the notion that degenerative discs always accelerate cellular senescence which may precipitate the pathology of IDD [4]. Disc cells undergo not only apoptosis but also dysfunction in IDD in an age-related manner. The latter, an abnormal cell state, plays a crucial role in matrix homeostasis imbalance. New pharmacological strategies focus on the elimination or reverse of senescent cells in degenerative discs for the prevention and treatment of IDD [5, 6].

As a fundamental cause of aging, cell senescence has been proved in all major chronic diseases in the cardiovascular system, nervous system, and especially musculoskeletal system and in chronic tumor [7–10]. However, the initial factors triggering disc cell senescence are exceptionally complex. Among the perspectives proposed in recent years to explain the emerging senescent cells in degenerative discs, one states that inner age-related stress and external microenvironment-derived stimuli both act as a promoter of cellular senescence to accelerate IDD [11]. Since the IDD is involved in multiple risk factors, mechanisms underlying these stressors that induce adaptive cell state changes have not been fully clarified. Finding critical intermediators from intricate cues seems to be conducive to inhibit cell senescence at the beginning of IDD.

In this review, we introduce the features of cell senescence and emphasize that it occurs as a general stress response. And we illustrate the impact of senescence on IDD. The role of oxidative stress and epigenetics linking multiple risk factors to cell senescence is summarized. Finally, we discuss relative therapeutic strategies in IDD. Hopefully, the brief introduction could primarily establish a link between cellular survival stress and IDD from a perspective of cell senescence.

## 2. Cell Senescence

### 2.1. The Features of Cell Senescence

Cell senescence is characterized by a cell state of proliferating arrest and secreting senescence-associated secretory phenotype (SASP) [12]. Cell cycle arrest plays a bilateral role in pathophysiological processes. On the one hand, it inhibits cell division and blocks the tissue renewal. On the other hand, it also prevents a further proliferation of harmful cells undergoing senescence [13]. Underlying cell cycle arrest, various molecular signals and pathways organize a complex network to exert effects [14]. All of them eventually converge on the p53/p21/retinoblastoma (RB) and p16/RB pathways to prevent senescent cell proliferation [14, 15]. Telomere shortening and stressor induction lead to replicative-related senescence (RS) and stress-induced premature senescence (SIPS) with respective mechanisms [16]. Previous investigations proved that these two major senescence phenotypes were involved in most chronic diseases.

As another major characteristic of senescent cells, SASP contains secretions of proinflammatory factors, chemokines, cytokines, protein enzymes, and other bioactive factors [17]. In mechanism, some certain pathways including nuclear factor kappa-B (NF-*κ*B), cyclic GMP-AMP synthase/stimulator of interferon genes (cGAS/STING), and mammalian target of rapamycin (mTOR) have been proved to control SASP [18, 19]. Meanwhile, epigenetic perturbations may regulate SASP at the transcriptional level [18]. This specific phenotype mediates cell-to-cell and cell-to-matrix signals to trigger immune-related clearance, inflammation formation, cell reprogramming, and microenvironment remodelling, which are involved in a series of diseases [17, 20–22]. Similar to senescence-associated markers like senescence-associated *β*-galactosidase (SA-*β*-gal), several major SASP components are used to indicate cell senescence [18, 23, 24]. However, the secretory factors are always variable due to their heterogeneity and nonspecificity. Therefore, increasing studies based on the diversity of SASP are carried out in recent years.

**Figure 1:**
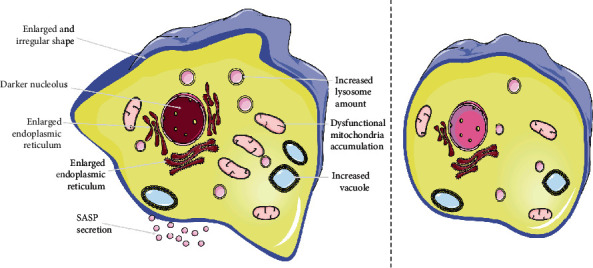
Morphological differences between senescent cells and normal cells. There are characteristic morphological alterations emerging in senescent cells compared with normal cells, such as increased but irregular cell size, darker nuclear chromatin, increased lysosome amount, dysfunctional mitochondrial accumulation, enlarged endoplasmic reticulum, increased vacuole, and secreting SASP. These features can be meaningful tools for identifying senescent cells and determining the senescent process.

In addition to cell cycle arrest and SASP, there are multiple changes of cellular components and morphology emerging in senescent cells [18] (Figure 1). These traits can be meaningful tools for distinguishing senescent cells and determining senescence process [4, 25–27]. However, cellular senescence is actually weakly defined due to the diversity of senescent biomarker expression in various subtypes. Recently, Ogrodnik et al. proposed a new model coordinating cell expansion and cell cycle arrest to screen senescent cells, which provided us a novel idea [28]. Meanwhile, increasing senescence subtypes and hallmarks are found in different cell types [18]. Exploring novel biomarkers with specifically differential expression in various senescence types is of great significance, which has become the focus of current researches [29, 30]. Although the standard of identification is not fully unified, antagonizing the progression of cell senescence has been a potential strategy in approaches to blunt chronic diseases.

### 2.2. Cell Senescence Occurs under Various Stressors

Cell senescence has been proved in majority of cell types as a response to various stressors [31]. The telomere exhaustion was firstly proved to cause cell senescence during cell division [32]. In addition to deoxyribonucleic acid (DNA) replication-associated stress, cells also face many other stressors from the inner nucleus such as oncogene activation [33]. For instance, the expression of oncogenic *ras* transforms rodent cells to a permanent G1 arrest by upregulating p53 and p16 [33]. The cell cycle arrest prevents the original tumorigenesis. Moreover, under the stress of radiotherapy [34] or chemotherapy [35] without a fatal dose, cells suffer DNA damage and are driven into the premature senescence instead of apoptosis to seek survival. Moreover, under stressful microenvironment, epigenomic perturbations also regulate senescence process. The modification of global or local chromatin alters specific gene expression to trigger cellular senescence independent of the DNA damage response (DDR) [36].

Meanwhile, cells need to withstand stress from the extra nucleus. As a cellular response to tissue damage, cells at the damage site undergo transient senescence. Senescent fibroblasts and endothelial cells secrete platelet-derived growth factor AA (PDGF-AA) to induce myofibroblast differentiation at the site of injury, ensuring wound closure [37]. Interestingly, senescent cells provoke plasticity of neighboring cells to enter transient reprogramming, which enhances differential potential and is conducive to tissue repair [38, 39]. The different role of senescent cells exists under embryonic development pressure. A p21-mediated senescence is found in the apical ectodermal ridge (AER). Growth factors like fibroblast growth factors (FGFs) are secreted to drive limb patterning and optimize tissue remodelling, which is recognized as a programmed senescence [40, 41]. Cells also encounter the stress from intracellular bioenergetic metabolism due to increasing dysfunctional mitochondria during aging [42]. As a metabolically active cell state, mitochondrial dysfunction-associated senescence (MiDAS) was proved to be implicated in this cell state transformation via adenosine 5′-monophosphate-activated protein kinase- (AMPK-) mediated p53 activation [42]. Despite the different stressors and complex signal pathways, the passive cell fate eventually arrives at the senescence outcome.

## 3. Cell Senescence Influences IDD

**Figure 2:**
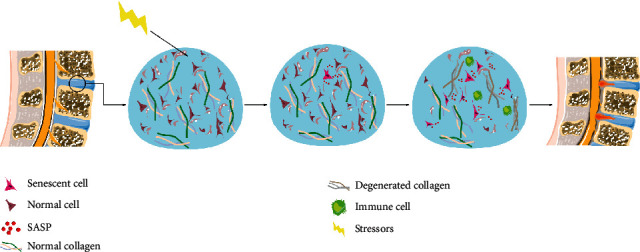
The role of cell senescence in the pathogenesis of IDD. Cell senescence occurs in response to intrinsic and extrinsic stressors in intervertebral discs. The primary senescent disc cells cause chronic inflammations, immune cell recruitment, and ECM degeneration through SASP. The deterioration of tissue microenvironment and persistent stress cause the accumulation of senescent cells which further accelerates tissue remodeling and eventually leads to IDD.

The intervertebral disc is composed of an inner highly hydrated colloid named the nucleus pulposus (NP) encircled by a dense fibrous ring, the annulus fibrosus (AF). The above parts are interfaced with the adjacent vertebrae through the vertebral endplates (EPs) [43]. There are specific features of oligocells and abundant matrix in intervertebral discs compared with other tissues like nervous or myocardial tissues [44]. Resident cells ensure long-term matrix renewal to maintain the balance of extracellular matrix (ECM) anabolism and catabolism with the huge survival burden during aging. Disc cells with slow turnover are suggested to be chronic senescence prone before apoptosis under cell replication and other stressors (Figure 2). A detrimental role of persistent senescent cells is predominant in chronic senescence processes like IDD [16, 33, 38]. Contrary to effective self-clearance of senescent cells in other tissues, the avascular nature of discs partly limits the immune-mediated clearance to cause the abnormal accumulation [11, 45]. Therefore, cell senescence in IDD results from not only the general aging process but also the structure and tissue specificity of discs.

### 3.1. Senescence Influences Functional Cells in IDD

As the functional cells in discs during intervertebral disc growth, NP, AF, and EP cells are in charge of ECM anabolic balance in separate regions, which play a key role in maintaining normal extracellular microenvironment [43]. These functional cells derived from degenerative discs all undergo the decline of proliferating potential, with high expressions of SA-*β*-gal and p16 (Ink4a) as well as decreased telomere length [4, 46, 47]. Interestingly, these senescence-associated changes in EPs seem to appear later than in AF and NP [46]. Relatively rich nutrition as well as milder local mechanical stress may explain this temporal sequence [48]. Although the cellular turnover in discs is slow, cell division during an expected lifespan of several decades is sufficient for inducing telomere length erosion and DNA damage to cause replicative stress. Subsequent activation of the p53/p21/RB pathway drives functional cells into the senescent cell state of cell cycle arrest [49]. The increasing stagnation during aging depletes the reservoir of cell renewal to further decrease remaining functional cells in IDD. Recent study found that deleting cell cycle inhibition gene p16 of NP cells could significantly decrease the proportions of senescent cells and SASP in degenerative discs [50]. Interestingly, Novais et al. observed the opposite result. They found that deficiency of p16 (Ink4a) did not alter the disc cell viability and the percentage of senescent cells [51]. Probably because p16 (Ink4a) acted as a main trigger for SIPS, knocking out this gene failed to avoid the replicative stress during degeneration [52, 53]. Despite the impotent inhibition of age-dependent degeneration, significant changes in the content and constituent of ECM occurred indeed. Changing senescent cells via targeting cell cycle relative genes or molecules has been focused on increasingly for ameliorating IDD.

In addition to replicative stress, as shock absorbers, the NP always suffers more mechanical stress than other tissues [54]. The accumulation of senescent NP cells was observed in scaffold and organ cultures under high-magnitude compression, confirming that mechanical-related SIPS existed in IDD [55]. Selective elimination of senescent NP cells protects tail suspension- (TS-) induced IDD mice from disc degeneration. Similar relationship between mechanical stress and cell senescence is proved in AF and EP cells [46, 47]. Further investigation indicated that the increased oxidative stress acted as a causative intermediator during mechanical factor-associated SIPS in degenerated discs [50]. In fact, intervertebral discs also have to face systemic stress derived from other diseases or risk factors. A critical review analysis indicated that vertebral EP capillaries got significantly narrower to block the nutrition supplement in diabetic animals, which might partly accelerate the diabetic-related IDD [56]. In diabetic patients, dysfunctional endothelial cells could exhibit senescence-like cell state via thrombospondin 1-cluster of differentiation 47- (TSP1-CD47-) dependent signaling to impair microvessels [57]. Although similar research in IDD is not reported, an endothelial senescence-related microcirculatory disturbance may partly promote the degeneration of EPs or even the whole discs under systemic risk factors like diabetes and hyperlipidemia. Therefore, cellular senescence in degenerative discs cannot always be ascribed to DNA replication and cell division as many cases of IDD occur in the stressful environment. In fact, RS and SIPS jointly accelerate the emergence of senescent cells in IDD compared with age-matched normal discs [4, 58].

### 3.2. Senescence Influences Extracellular Microenvironment in IDD

In addition to the stagnation of cell proliferation, the senescent disc cells also overexpress a range of bioactive components as fundamental features of SASP [12, 59–62]. SASP during IDD plays a crucial role in altering tissue microenvironment [12, 63]. Among them, proinflammatory factors such as interleukin-1*β* (IL-1*β*), interleukin-6 (IL-6), interleukin-8 (IL-8), and tumor necrosis factor-*α* (TNF-*α*) have been proved to be generally upregulated by senescent cells to trigger inflammatory cascades, which cause the chronic inflammation and accelerate IDD [51, 64]. Despite the low specificity, growing studies recognize them as indexes to cell senescence [18, 23, 24]. Apart from the inflammatory effect, some specific cytokines like IL-6 and IL-8 also provide intercellular dialogue to induce adjacent cells to be senescence or reprogramming prone [12, 21, 24, 39]. The persistent dedifferentiation of neighboring cells may inhibit the supplement for lost or damaged cells and disturb tissue rejuvenation [65]. Meanwhile, specific SASP like (C–C motif) ligand 2 (CCL-2), vascular endothelial growth factor (VEGF), transforming growth factor *β* (TGF-*β*), and chemokine (C–C motif) ligand 20 (CCL-20) are proved to induce adjacent normal cells to be “paracrine senescence” [63]. Although it has not been fully clarified in IDD, these cell-to-cell signals may partly promote the accumulation of dysfunctional disc cells.

As the fertile soil for NP cells, collagen matrix consisted of generous type II collagen and aggrecan provides nutrients for cell metabolism and maintains disc tensile stiffness [43]. Collagen falls into the stagnation period of renewal during the development of IDD. Che et al. observed that many matrix remodelling enzymes in SASP like matrix metalloproteinases (MMPs) family (MMP3, MMP9, MMP10, and MMP13) were secreted increasingly in senescent disc cells of TS mice [66]. MMPs degraded collagen II as well as ECM proteins like proteoglycan and aggrecan, decreasing hydration of NPs in IDD. In addition to MMPs, senescent disc cells also raise the level of a disintegrin and metalloproteinase with thrombospondin motifs (ADAMTS) family (ADAMTS-4, ADAMTS-5). The increased proteolytic enzymes cleave the aggrecan interglobular domain and drive the matrix PG loss in vitro compared to nonsenescent disc cells, where certain pathways like *β*-catenin may play a key role [62, 67, 68]. As a crucial regulator in joint development, *β*-catenin is proved to mediate cell senescence in cartilages and discs, indicating that analogical cartilaginous tissue may be comparable in senescence mechanism [68, 69]. The continuous secretion of catabolic enzymes produced by senescent cells severely perturbs the ECM homeostasis since disc cells are unable to act as the major producer of ECM. Notably, SASP in senescent disc cells may be a unique response of tissue to different stress like physical activities, and the specific disc structure for low pH, low oxygen tension, and cellular environment may further amplify this effect of SASP on ECM [12, 70].

## 4. The Underlying Pathogenesis of Cell Senescence in IDD

As a multifactor-induced degenerative disease, IDD is associated with many potential risk factors such as abnormal mechanical loading, inappropriate exercise, obesity, and even specific diet style [55, 71, 72]. Growing studies indicate that oxidative stress (OS) and epigenomic perturbations may act as major mediators between these risk factors and cell senescence in IDD [73].

### 4.1. Oxidative Stress

**Figure 3:**
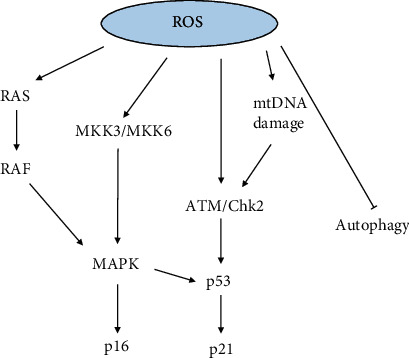
The signaling pathways between ROS and cell senescence.

Reactive oxygen species (ROS) production is inevitable due to the oxygen-utilizing metabolism of disc cells despite the hypoxic microenvironment in IDD [74]. Subsequent OS increases cellular survival stress which activates the DDR to trigger cell senescence and force the dysfunction of resident cells [45, 75]. Apart from the hasher microenvironment like insufficient nutrient supply and metabolite clearance during aging, exogenous stimuli exacerbate excessive ROS generation in disc cells [76]. These excessive oxidizing products drive disc cells into the state of SIPS in IDD [14]. Underlying the cellular transformation, diverse signaling pathways have been proved to participate in the senescence process (Figure 3). Considering the nonmitochondrial-dependent ROS productions like nicotinamide adenine dinucleotide phosphate (NADPH) oxidase and xanthine oxidase are scarcely reported in the discs, we focus on the role of mitochondrial-derived ROS in cell senescence during IDD [76, 77].

NP cells exhibit the stagnant state with senescence markers like high SA-*β*-gal activity by the accumulation of ROS in NP cells under various stimuli such as high-magnitude compression [4] and high glucose [58]. Indeed, the OS appears due to the imbalance between excessive ROS generation and impaired antioxidative molecules or enzymes disturbed by these stressors [78]. Recent studies significantly inhibited cell senescence through reversing this imbalance, emphasizing the role of OS in degenerative discs. The hydrogen peroxide (H_2_O_2_) was used to aggravate intracellular oxidative stress to provoke DNA damage and successfully induced cell cycle arrest as well as SASP of NP cells via the ataxia telangiectasia-mutated checkpoint kinase 2-p53-p21 (ATM-Chk2-p53-p21) pathway [58, 79]. This H_2_O_2_^−^-induced premature senescence could be partly reversed by the antioxidants like N-acetyl-L-cysteine [58]. Targeting the elimination of ROS components like H_2_O_2_, hydroxyl (·OH), and superoxide anion (·O_2_^−^) becomes a potential therapeutic strategy. Recently, Larrañaga et al. constructed antioxidant polymer capsules, which can be englobed by NP cells to scavenge intracellular ROS like H_2_O_2_ and ·OH. The decline of ROS level significantly attenuated the catabolic and proinflammatory cell phenotype [80]. In addition, the ROS-induced autophagy inhibition is also proved to cause oxidative stress in many cell types [81–83]. And by enhancing and restoring impaired autophagy during IDD, Kang et al. affirmed that excessive ROS production could be suppressed to protect the mitochondrial hemostasis and ameliorate NP cell senescence [84].

Mitochondria are not only the main source of excessive ROS production but also the major attacking target of ROS [37, 76, 85, 86]. Literature demonstrated that OS could be increased due to proinflammatory factors like TNF-*α* to induce mitochondrial dysfunction to aggravate the senescence in NP cells [87, 88]. Moreover, TNF-*α*-induced abnormal OS can also disturb hypoxic conditions in senescent EPs, which transfer the cartilage endplate stem cell (CESC) differentiation from chondrogenesis to osteogenesis [89]. The ROS dysregulate respiratory enzyme activity and cause the respiratory chain defect in dysfunctional mitochondria, leaking extensive electrons to produce more ROS [76]. Lack of effective antioxidant mechanisms during senescence, a ROS-mediated positive feedback loop may act as a critical murderer to accelerate senescence. In turn, interventions targeting cellular senescence may attenuate the oxidative stress. For instance, Che et al. observed a reverse of senescent NP cells by p16 deletion in IDD, which was companied with a reduced oxidative stress [50]. A strong interaction between oxidative stress and disc cell senescence brings us more possibility for intervening IDD. Moreover, Wang et al. confirmed that ROS-mediated mitochondrial dysfunction could be inhibited by polydatin via activating the Nrf2/hemeoxygenase 1(HO-1) pathway to prevent senescence in rat NP cells [88], further testifying a significant effect of blocking this vicious circle. Although other pathways independent of ROS may also mediate the mitochondrial dysfunction to drive the senescent phenotype [85], maintaining a mitochondrial homeostasis should exert antioxidative benefits on preventing cell senescence.

### 4.2. Epigenomic Perturbations

Senescence phenotypes always appear in various forms no matter in different senescent cells or different senescence stages of the same cell [90]. Multiple stressors can be drivers of disc cell senescence through altering chromatin structural organization or gene expressions during aging, where epigenetic perturbations act as a critical link [91].

In recent years, increasing studies have focused on the role of epigenetics during IDD. As major protein components of chromatin, histone and related modifications participate in DNA damage-induced senescence. Under various stressors, the histone phosphorylation at the site of DNA damage initiates the first step of cascade reactions from DDR to the activation of p53/p21/Rb and eventually causes an irreversible cell cycle arrest [60, 92, 93]. The histone trimethylation also plays a critical role in the positive feedback loop between enhancer of zeste homolog 2 (EZH2) and NADPH oxidase 4 (NOX4) to promote the NP cell senescence during IDD [94]. The latter of the loop has been proved as an important mediator of various stressors to induce OS-associated SIPS in degenerative discs [94]. Recently, Williams et al. observed a hypermethylation level at one cytosine guanine (CpG) site (cg15832436) of the parkinson protein 2 (PARK2) promoter in IDD [95]. The modification of PARK2 at transcriptional level may regulate mitochondrial-associated OS in degenerative NP and EP cells via parkin-mediated mitophagy [96, 97]. Therefore, epigenetic changes may affect cellular senescence via the regulation of OS at transcriptional level. In addition, the genome-wide analysis of DNA methylation profile indicated that DNA methylation profiles were significantly distinct between early and advanced stages of human IDD, especially in NP tissue [98]. The group further identified 220 differentially methylated loci comprising 187 individual genes. Among them, Wnt-5a, one of the representative ligands of the wingless/integration-1- (Wnt-) *β*-catenin pathway, was observed differentially methylated in advanced stage of IDD [98]. Notably, the Wnt-*β*-catenin pathway has been reported to be relative to promote disc cell senescence via a positive feedback loop of Wnt signaling and cytokines [60]. Moreover, they also found several hypermethylated genes that may play important roles in regulating catabolic molecules of IDD, such as caspase recruitment domain 14 (CARD14), EF-hand domain-containing protein D2 (EFHD2), rhotekin 2 (RTKN2), mitogen-activated protein kinase-activated protein kinase 5 (MAPKAPK5), and protein kinase C/*ζ* (PRKCZ) associated with the MAPK pathway [98]. These mitogen-activated protein kinase (MAPK) and NF-*κ*B-associated genes may be involved in controlling SASP. The differential methylations dominantly exist in genes which are located upstream instead of directly regulating downstream target genes associated with proinflammatory cytokines and catabolic molecules [98]. However, considering a series of discoveries about DNA demethylation located in promoter sites of catabolic enzyme MMPs in osteoarthritis (OA), further studies like ribonucleic acid (RNA) sequencing are needed to explore this possibility in IDD with more samples [99].

Based on previous literature, the overexpressions of many catabolic enzymes like MMPs and ADAMTS are recognized as characteristics of senescence-associated ECM remodelling in senescent discs during IDD [50, 62]. Many microRNAs have been proved to target these enzymes to modulate the ECM remodeling process through a specific pathway, respectively [100–102]. Notably, some of them such as microRNA-132 (miR-132) and microRNA-494 (miR-494) are reported to be upregulated by the methylation of CpG islands in the promoter region in degenerative human NP cells [103, 104]. Secreted protein acidic and rich in cysteine (SPARC) is widely accepted as another matricellular protein to regulate cell-matrix interactions and collagen fibrillogenesis in IDD [105]. And the hypermethylation of SPARC promoter is observed to decrease related gene expressions of degenerative discs in both humans and animal models, which may partly cause ECM remodeling in IDD [106].

Although the relationship between epigenetics and cell senescence in IDD has been primarily established, how various stressors trigger these epigenetic perturbations is still unclear. Further exploration for these epigenetic changes will provide us more potential biomarkers as well as therapeutic targets for the management of IDD (Table 1).

## 5. The Promising Therapy for IDD: Senotherapies

Optimizing disc health in an aging population requires maintaining normal renewal and proliferation as well as function of parenchymal cells [107]. Due to the key role that cell senescence plays in IDD, recent studies highlight the significance of developing senotherapeutic approaches that target senescent cells therapeutically to blunt or even reverse IDD.

There are several potential strategies for antisenescent cells including directly eliminating senescent cells, targeting SASP for reducing its adverse effects, and relieving senescence-associated growth arrest [108]. The first way seems to be the most direct and hitting home. Specifically, preventing accumulation of p16(Ink4a)-positive senescent cells in intervertebral discs has been proved to ameliorate matrix homeostasis and mitigate the progress of IDD [51, 109]. Furthermore, small molecules termed senolytics have emerged in recent years, and these selective eliminators of senescent cells are considered to exert great benefits on antiaging process [110]. Dasatinib and quercetin targeting ephrin (Eph) receptor tyrosine kinases and phosphatidylinositol 3-kinase/protein kinase B (PI3K/AKT) pathway modules, respectively, were the first molecules to be identified as senolytics in 2015 [5]. From then on, many drugs are found to be efficacious in animal models and human cells or tissues [73]. Some known antiaging compounds against other diseases exert new senotherapeutic activity on IDD. As an antidiabetic drug before, metformin confers antisenescence effect in NP cells and annulus fibrosus stem cells (AFSCs) which significantly ameliorate disc degeneration [111, 112]. Classic bisphosphonate antiosteoporosis (OP) drug has been demonstrated to benefit not only OA but also IDD [6]. The discs of rats treated with alendronate significantly retarded EP thickening and increased the aggrecan and type II collagen anabolism in discs [6]. Although the underlying mechanism has not been clarified, the analogical benefits in OP, OA, and IDD provide the possibility for preventing the whole musculoskeletal system aging and degeneration. Classic senotherapeutic drug repurposing emerges as an interesting aspect and may provide potential appealing therapeutic approaches for IDD [110]. Notably, whether the excellent result is a cause of antiaging effects or a consequence is worth considering because of the multifaceted effects of these compounds [12]. Despite their senotherapeutic effects, these drugs possess various disadvantages such as off-target effect and low potency. For example, the usage of dasatinib is reported to inhibit hypoxic pulmonary vasoconstriction to induce pulmonary hypertension independent of regular Src kinase and tyrosine kinase-induced mechanism [113]. As a broad-spectrum kinase inhibitor, it may target potential irrelevant kinases to cause nonessential damage [12]. Moreover, the avascular structures in intervertebral discs are not conducive to drug accumulation after systemic administration, which may further amplify the off-target effect and low potency. Therefore, delivering drugs directly to degenerative sites may partly avoid these deficiencies. In recent years, some drug delivery systems connected with antibodies against senescence-specific surface antigens are synthesized to solve the problem of targeted drug delivery. Qu et al. used the beta-2-microglobulin antibody (anti-B2MG) to successfully construct a nanoparticle tetrahedron which could recognize and selectively clear senescent cells [114]. Amor et al. screened a cell surface protein called the urokinase-type plasminogen activator receptor (uPAR) and constructed senolytic chimeric antigen receptor (CAR) T cells to eliminate target cells in multiple senescence models, which emerged as a striking senolytic strategy [29]. Therefore, further screening and identifying senescence-specific surface antigens will provide more potential targets, which may facilitate the diagnosis and interventions of senescence [29, 115].

Another promising strategy is targeting the SASP for blocking the adverse effects of specific components like proinflammatory factors and matrix catabolic enzymes, without changing the inner antioncogenic pathways and cell cycle in senescent cells [108, 116]. This could be achieved by targeting signaling cascade upstream of the SASP of senescent disc cells, such as NF-*κ*B or p38 MAPK as well as related regulators [12, 87, 117]. For instance, depletion of ATM downregulates the expression of SASP factors like ADAMTS and MMPs and significantly attenuates age-associated IDD [118, 119]. However, the SASP expression on different cells, tissues, and organs is not always constant considering the diversity of senescence types [65]. Finding relative conserved molecular phenotypes existing in different senescence types will be meaningful for clarifying the mechanism underlying SASP and find potential targets [65]. Moreover, considering the self-clearing mechanism of senescent cells via SASP [120–122], it is essential to weigh the benefit between SASP-mediated immune clearance and inflammation damage in IDD and the conclusion is still not be casually established [12]. To avoid this dilemma, targeting specific proinflammation factors or proteins like TNF-*α*, MMP instead of the whole SASP is considered to be more promising for anti-IDD [43, 123]. Therefore, accurately identifying “the black sheep” of SASP and relative signal pathways in disc senescence may develop SASP-related interventions further.

The third approach, relieving senescence-associated growth arrest, could be realized through blocking the effect of cell cycle inhibitor like p16 and forkhead box O4 (FOXO4) or deleting relative genes [50]. Baar et al. synthesized a competitive inhibitor of FOXO4 to perturb the interaction between p53 and FOXO4 and successfully transferred the cell cycle from arrest to apoptosis [124]. However, this strategy needs to be treated with caution, considering that a potential excessive cell replication and proliferation may trigger tumorigenesis [108]. In contrast, reserving the senescent state instead of directly eliminating them seems to be more appealing because of the rare cell number in degenerative discs. There is no relative report in IDD, but Zhang et al. realized the reprogramming of senescent muscle stem cells via supplying cellular nicotinamide adenine dinucleotide (NAD^+^) and increased mouse lifespan [125]. Regardless of the difference of cell characteristics, restoring the function of senescent disc cells will greatly ameliorate the condition of oligocytes and cell dysfunction in degenerative discs.

## 6. Prospects of Cell Senescence in IDD

In this review, we demonstrated the pathogenesis of disc degenerative diseases from a perspective of cell senescence and highlighted the pivotal role of oxidative stress and epigenomic perturbations played. And we analyzed the potential of interventions targeting senescent cells and the SASP, which seemed to be prospective strategies. Notably, there are some confusing questions to be answered: one unsolved is that most applications of current senescent animal models inevitably ignore the accumulation of time effect. And it is unconvincing to attribute the degeneration entirely to cell senescence, considering the real chronic degeneration process in our body. In addition, the heterogeneity of cell senescence in different cells or tissues has been proved, but little is known about that which may exist in the same cell types in intervertebral discs. Investigating every single senescent cell could be an interesting topic, which may help us further understand the detail of cell senescence in IDD. Finally, days are early to say whether changing the outcome of senescent cells can really ameliorate or even reverse the progression of disc degeneration, as many emerging strategies are only preliminarily proved in vitro. Cell senescence itself is a complex biological process that has not been fully clarified, and more problems could be readily solved when further cognition is established.

## Figures and Tables

**Table 1:** Function characterization of epigenetic alterations in IDD in text.

Name	Type	Target	Expression	Functional role	Reference
/	Histone phosphorylation	Chk2	Up	Cell cycle arrest	[92, 93]
H3K27me3	Histone trimethylation	NOX4	Up	Oxidative stress	[94]
PARK2	DNA methylation	Parkin	Up	Mitophagy	[95, 96]
WNT5A	DNA methylation	Wnt-*β*	Up	Proinflammation	[98]
CARD14	DNA methylation	NF-*κ*B	Up	Proinflammation	[98]
EFHD2	DNA methylation	NF-*κ*B	Up	Proinflammation	[98]
RTKN2	DNA methylation	NF-*κ*B	Up	Proinflammation	[98]
MAPKAPK5	DNA methylation	MAPK	Up	Proinflammation	[98]
PRKCZ	DNA methylation	MAPK	Up	Proinflammation	[98]
miR-21	Noncoding RNA	PDCD4	Down	Proliferation	[100]
miR-146a	Noncoding RNA	TRAF6	Down	ECM degradation	[101]
miR-98	Noncoding RNA	IL-6/STAT3	Down	Inhibition of ECM degeneration and apoptosis	[102]
miR-494	Noncoding RNA	SOX9	Down	ECM degeneration and apoptosis	[103]
miR-132	Noncoding RNA	GDF5	Down	ECM degradation	[104]
SPARC	DNA methylation	SPARC	Down	ECM degradation	[106]

## Supplementary Materials

Supplementary Materials The supplementary material is a summary list of abbreviations and their corresponding full names mentioned in this review.
