# Supplementary material for: Cell Senescence: A Nonnegligible Cell State under Survival Stress in Pathology of Intervertebral Disc Degeneration
Source: Oxid Med Cell Longev. 2020 Aug 31;2020:9503562. doi: 10.1155/2020/9503562 (PMC7479476; doi:10.1155/2020/9503562)
Supplement: Supplementary Materials — The supplementary material is a summary list of abbreviations and their corresponding full names mentioned in this review. [file 9503562.f1.pdf]

Supporting Information

Cell Senescence: A Nonnegligible Cell State under Survival Stress in Pathology of Intervertebral Disc Degeneration

Yuang Zhang, [1,2] Biao Yang,[1,2] Jingkai Wang,[1,2] Feng Cheng,[1,2] Kesi Shi,[1,2] Liwei Ying,[1,2] Chenggui Wang,[1,2] Kaishun Xia,[1,2] Xianpeng Huang,[1,2] Zhe Gong,[1,2] Chao Yu,[1,2] Fangcai Li,[1,2] Chengzhen Liang,[1,2] and Qixin Chen[1,2]

[1]Department of Orthopedics Surgery, The Second Affiliated Hospital, Zhejiang University School of Medicine, 310009 Hangzhou, Zhejiang, China.

[2]Zhejiang Key Laboratory of Bone and Joint Precision and Department of Orthopedics Research Institute of Zhejiang University, Hangzhou, Zhejiang 310009, China.

Correspondence should be addressed to Fangcai Li; lifangcai@zju.edu.cn or Chengzhen Liang; liangchengzhen@zju.edu.cn or Qixin Chen; zrcqx@zju.edu.cn

Contents

1. The List of Abbreviations

Abbreviation	
IDD	intervertebral disc degeneration
LBP	low back pain
YLD	years lived with disability
SASP	senescence associated secretory phenotype
RS	replicative-related senescence
SIPS	stress-induced premature senescence
SA-β-gal	senescence associated β-galactosidase
PDGF-AA	platelet-derived growth factor AA
AER	apical ectodermal ridge
MiDAS	mitochondrial dysfunction-associated senescence
ROS	reactive oxygen species
NP	nucleus pulposus
AF	annulus fibrosus
EPs	endplates
ECM	extracellular matrix
TS	tail suspension
PG	proteoglycan
CESC	cartilage endplate stem cell
OA	osteoarthritis
DDR	DNA damage response
H_2O_2	hydrogen peroxide
$\cdot O_2^-$	superoxide anion
·OH	hydroxyl
MMP	matrix metalloproteinase
ADAMTs	a disintegrin and metalloproteinase with thrombospondin motifs
OP	osteoporosis
anti-B2MG	beta-2-microglobulin antibody
RB	retinoblastoma
NF-kB	nuclear factor kappa-B
cGAS/STING	cyclic GMP-AMP synthase/ stimulator of interferon genes
IL-1	interleukin-1
IL-6	interleukin-6
IL-8	interleukin-8
mTOR	mammalian Target of Rapamycin
DNA	deoxyribonucleic acid
FGFs	fibroblast growth factors
AMPK	adenosine 5’-monophosphate-activated protein kinase

IL-1β	interleukin-1β
TNF-α	tumor necrosis factor-α
CCL-2	chemokine (C–C motif) ligand2
VEGF	vascular endothelial growth factor
TGF-β	transforming growth factor β
CCL-20	chemokine (C–C motif) ligand 20
TSP1-CD47	thrombospondin 1- cluster of differentiation 47
OS	oxidative stress
NADPH	nicotinamide adenine dinucleotide phosphate
ATM-Chk2-p53-p21	ataxia telangiectasia mutated-checkpoint kinase 2-p53-p21
Nrf2	nuelear faetor E2 related faetor 2
Keap1	Kelch-like ECH-Associating protein 1
HO-1	hemeoxygenase 1
NOX4	NADPH oxidase 4
PARK2	parkinson protein 2
Wnt	wingless / integration-1
CARD14	caspase recruitment domain 14
EFHD2	EF-hand domain-containing protein D2
RTKN2	Rhotekin 2
MAPKAPK5	mitogen-activated protein kinase-activated protein kinase 5
PRKCZ	protein kinase C/ζ
MAPK	mitogen-activated protein kinase
RNA	ribonucleic acid
miR-132	microRNA-132
miR-494	microRNA-494
SPARC	secreted protein acidic and rich in cysteine
H3K27me3	trimethylation of lysine 27 on histone 3
Chk2	checkpoint kinase 2
WNT5A	wingless / integration 5A
miR-21	microRNA-21
miR-146a	microRNA-146a
miR-98	microRNA-98
PDCD4	programmed cell death 4
TRAF6	TNF receptor associated factor 6
STAT3	signal transducer and activator of transcription 3
SOX9	Sry related HMG box 9
GDF5	growth and differentiation factor 5
Eph	ephrin
PI3K/AKT	phosphatidylinositol 3 kinase/protein kinase B
AFSCs	annulus fibrosus stem cells

FOXO4	forkhead box O4
NAD^+	nicotinamide adenine dinucleotide
uPAR	urokinase-type plasminogen activator receptor
CAR	chimeric antigen receptor
